# Managing gastrointestinal involvement in cutaneomucosal venous malformation: safety and efficacy of endoscopic sclerotherapy

**DOI:** 10.1186/s41065-025-00486-5

**Published:** 2025-06-23

**Authors:** Jiaxue Zhu, Wei Liu, Zehua Zhang, Bensong Duan, Xiaohan Yan

**Affiliations:** 1 Department of Interventional Therapy, Multidisciplinary Team of Vascular Anomalies, Shanghai Ninth People’s Hospital, Shanghai Jiao Tong University School of Medicine, Shanghai, P. R. China; 2 Endoscopy Center, Department of Gastroenterology, Shanghai East Hospital, School of Medicine, Tongji University, Shanghai, 200120 China

**Keywords:** Cutaneomucosal venous malformation, Endoscopic Sclerotherapy, TEK, Blue Rubber Bleb Nevus Syndrome

## Abstract

Blue rubber bleb nevus syndrome (BRBNS) and cutaneomucosal venous malformation (VMCM) both manifest as venous malformations (VMs) characterized by blue, compressible nodules. While BRBNS typically involves visceral organs, particularly the gastrointestinal (GI) tract, VMCM has conventionally been considered to spare internal organs. Our findings, however, reveal that VMCM can indeed extend to the GI system and demonstrate that endoscopic sclerotherapy is safe and effective for its management. This underscores the importance of genetic testing and systemic evaluation in patients with multifocal VMs, suggesting the need for revised diagnostic criteria and a more nuanced approach to classification of these disorders.

## Key points

• Cutaneomucosal venous malformation (VMCM), traditionally considered limited to skin, can involve the gastrointestinal tract, challenging current diagnostic classifications and necessitating systemic evaluation in patients with multifocal venous malformations.

• Endoscopic Sclerotherapy is effective and safe for managing colonic venous malformations in VMCM, suggesting its potential as a first-line local therapy for GI involvement, analogous to approaches in BRBNS.

## Introduction

Blue rubber bleb nevus syndrome (BRBNS) and cutaneomucosal venous malformation (VMCM) are both classified as venous malformation (VM) disorders, but they exhibit significant differences in genetic mechanisms, clinical manifestations, and lesion distribution [1].

BRBNS is a multisystem disorder driven by somatic double mutations in TEK [1], while VMCM is an inherited disease caused by germline TEK mutations requiring a second somatic hit for lesion formation [1, 2]. Both conditions involve aberrant PI3K/AKT pathway activation, making them potentially responsive to targeted therapies such as rapamycin (sirolimus) [1, 3].

BRBNS has been documented as a syndrome with predominant gastrointestinal (GI) involvement, leading to chronic hemorrhage and anemia. Multiple case reports emphasize that BRBNS lesions commonly affect the GI tract, particularly the small intestine, causing occult or overt bleeding that manifests as iron deficiency anemia requiring frequent transfusions [4–6]. This syndrome often involves skin, but GI lesions are prevalent in up to 89% of patients, contributing to long-term morbidity such as anemia due to ongoing blood loss [7–9]. In contrast, VMCM has traditionally been perceived as a condition limited to cutaneous and mucosal areas, without substantial GI implications [1, 10]. This study presents genetically confirmed VMCM with significant GI involvement, challenging the conventional understanding of VMCM, thereby suggesting a broader phenotypic spectrum that necessitates re-evaluation of diagnostic protocols and comprehensive systemic evaluation prior to therapeutic intervention.

## Report

**Fig. 1:**
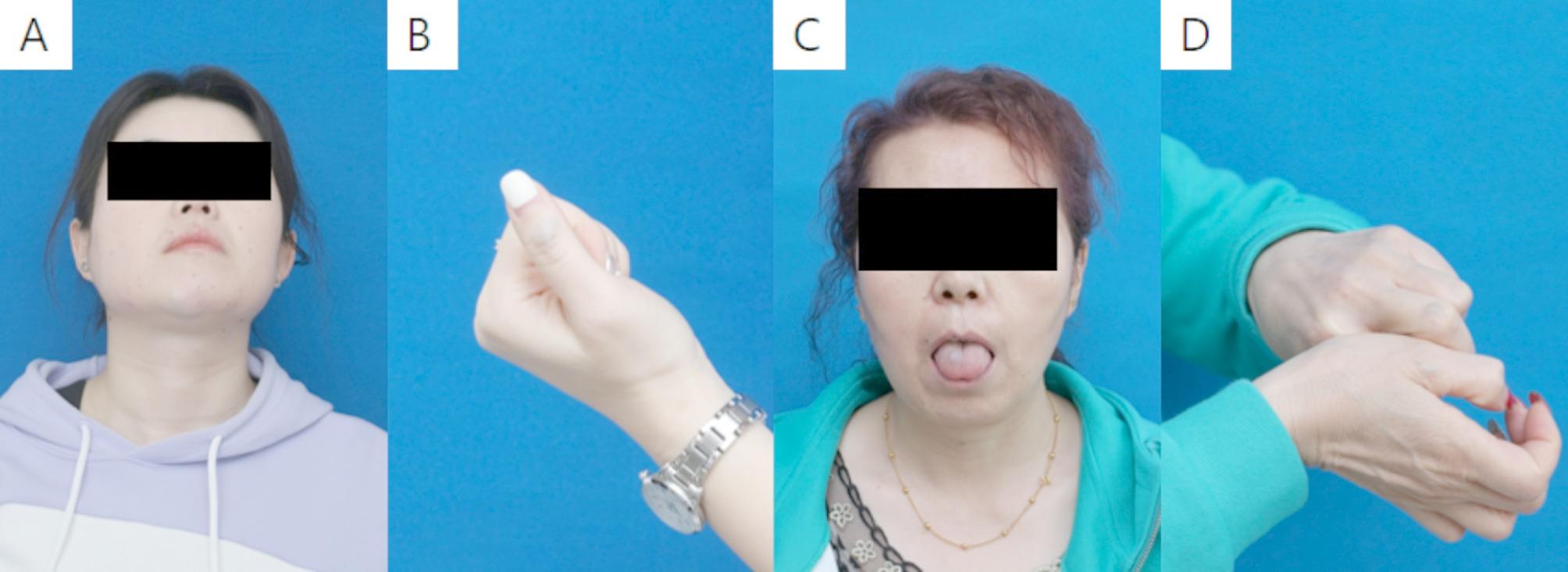
The clinical manifestation of the patient and her mother. **A**: The patient exhibited multiple bluish mucosal lesions on her face. **B**: Similar vascular formations on the right hand of the patient. **C**: Similar vascular formations on the lips and tongue of the patient’s mother. **D**: Similar vascular formations on the hands of the patient’s mother

A 28-year-old woman with anemia was referred to our clinic. Laboratory investigations revealed a hemoglobin (Hb) level of 105 g/L and a ferritin level of 13 µg/L. Beginning at 3 months postpartum, the patient developed multiple bluish vascular lesions on the facial skin, right hand, and right forearm. From 26 years of age, she experienced recurrent anemia with hemoglobin levels fluctuating around 108 g/L. Her mother also exhibited multiple bluish mucosal lesions on the tongue and lips and hands (Fig. 1c-d), without gastrointestinal involvement, diagnosed as VMCM. Physical examination demonstrated similar vascular formations on the facial skin, right hand, and right forearm (Fig. 1a-b).

**Fig. 2:**
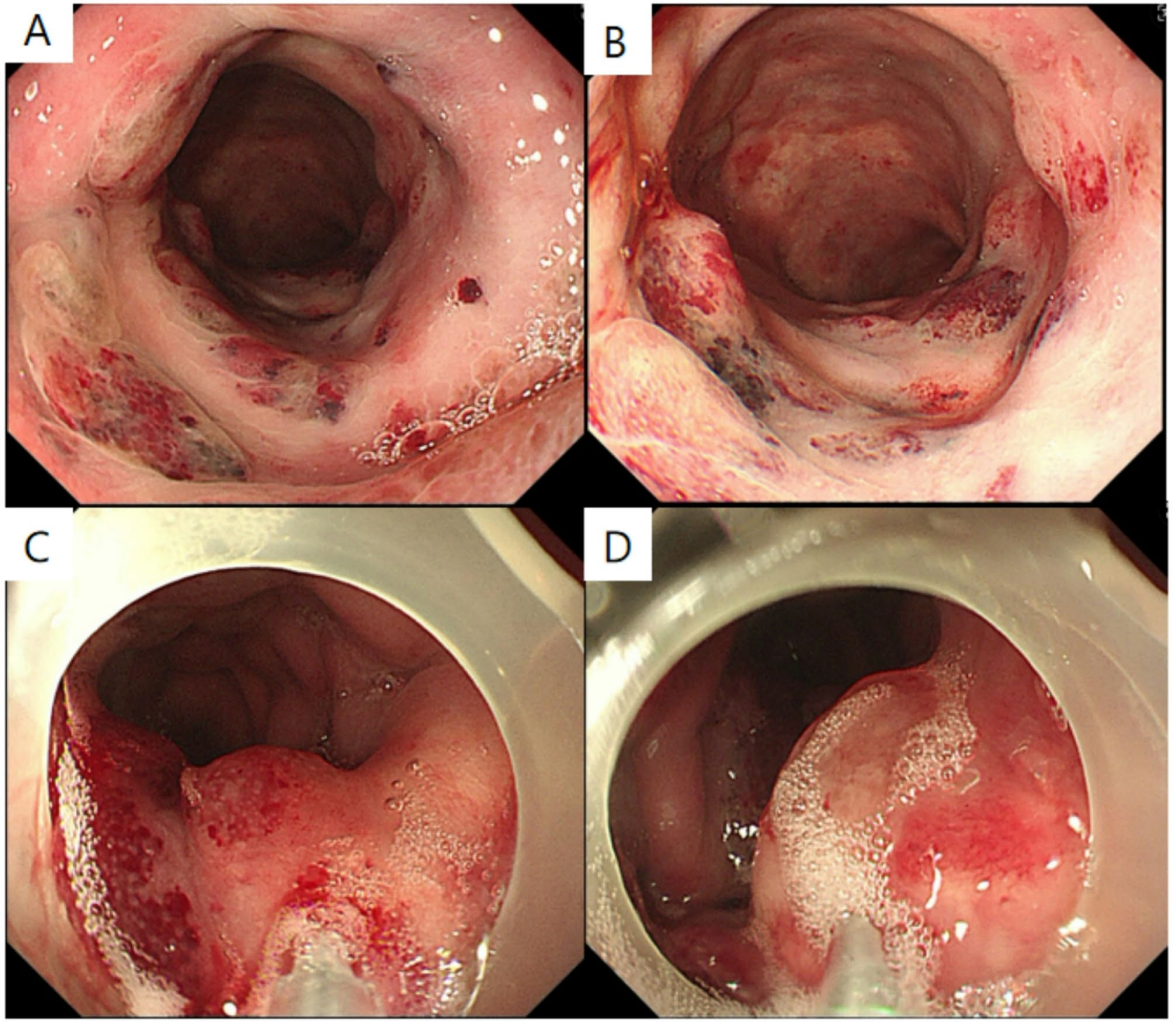
The endoscopic findings and the process of the colonoscopic sclerotherapy of the patient. **A**-**B**: Numerous bluish vascular formations in the colon. **C**-**D**: After colonoscopic sclerotherapy with polidocanol, the lesions turned white

Colonoscopy identified numerous bluish submucosal lesions in the transverse colon, ranging in size from 0.4 mm to 1.8 cm (Fig. 2a-b). Gastroscopy and capsule endoscopy showed no vascular malformations, active bleeding, or blood clots from the tongue, pharynx, esophagus, stomach, or small intestine. Specimen of peripheral blood and tissue lesion were sent for targeted Next-Generation Sequencing (NGS). Genetic study revealed TEK germline mutation of TEK (p.Arg849Trp) at frequency of 48.2% in peripheral blood and 47.6% in tissue. In the meanwhile, TEK(p.Arg849Trp) was also detected in the proband’s mother’s blood specimen. This patient was diagnosed as VMCM in clinical manifestation and genetic study. The patient is on a twice-daily oral sirolimus regimen, dosed at 0.8 mg/m² of body surface area per administration.

Under general anesthesia, the patient underwent colonoscopic sclerotherapy with polidocanol for the venous malformations in the transverse colon (Fig. 2c-d). Foam sclerosant (20 ml polidocanol + 10 ml air) was injected at around 10 separate sites, with approximately 1–2 ml of foam sclerosant administered per injection site. No procedure-related adverse events (e.g., postoperative hemorrhage, embolism, or perforation) occurred. During the two-year follow-up period, the patient remained clinically stable with no recurrence of anemia or melena.

## Discussion

The differentiation between BRBNS and VMCM is critical due to overlapping presentations but divergent management pathways. BRBNS is frequently associated with specific genetic mutations (e.g., TEK), and its GI involvement often leads to delayed diagnosis or misdiagnosis if cutaneous signs are absent [11]. Cases exist where BRBNS was undetected for years due to atypical symptoms, highlighting the importance of genetic testing in confirming the diagnosis [11]. Similarly, our patient’s genetic testing excluded BRBNS, confirming VMCM despite GI lesions. This underscores that patients with multiple venous malformations require thorough differential diagnosis involving gene testing to distinguish between syndromes like BRBNS (which typically involves GI) and VMCM (now shown to have GI potential). Routine gastroenteroscopy should be implemented prophylactically in such patients to identify asymptomatic or early-stage GI lesions, thereby preventing complications like chronic anemia. Anemia in this context is not uncommon, as refractory anemia often stems from GI bleeding in vascular malformation syndromes, emphasizing the need for prompt endoscopic investigation to mitigate iron deficiency and transfusion dependency [4, 5, 9, 12].

For management, localized treatment of skin lesions (e.g., resection or sclerotherapy) should be supplemented with GI surveillance and intervention. The BRBNS literature supports endoscopic approaches, such as endoscopic sclerotherapy, which has demonstrated efficacy in achieving hemostasis and preventing recurrence [13, 14]. While GI lesions in VMCM lack established management guidelines, endoscopic approaches validated in BRBNS (e.g., sclerotherapy) may be adaptable. In this study, the patient underwent successful colonoscopic sclerotherapy for colonic vascular malformations, with no recurrence observed during the 2-year follow-up period. This suggests that endoscopic sclerotherapy may represent an effective, safe, technically straightforward approach with rapid recovery for managing GI manifestations of VMCM. While sirolimus-based therapies have shown efficacy in BRBNS [7, 15], these systemic treatments should generally be reserved for extensive lesions, with endoscopic modalities remaining first-line for accessible GI lesions. Proactive GI screening following cutaneous treatment is crucial, as lesions may develop insidiously and progressively worsen anemia over time.

## Conclusion

This study has significant clinical implications: VMCM’s GI involvement represents a novel phenotypic variant for which endoscopic sclerotherapy proved safe and effective, urging updates to diagnostic criteria. Future research should explore the genetic underpinnings of GI affection in VMCM and standardize endoscopic surveillance protocols to improve outcomes.

## Data Availability

No datasets were generated or analysed during the current study.
